# Idiopathic pneumoperitoneum in a single case: an unusual entity

**DOI:** 10.1093/jscr/rjad330

**Published:** 2023-06-17

**Authors:** Oluwafolaranmi E Sodade, Cindy L Austin, Brian B Draper

**Affiliations:** Mercy Research—Trauma Research, Springfield, MO, USA; Mercy Research—Trauma Research, Springfield, MO, USA; Mercy—General & Trauma Surgery, Springfield, MO, USA

**Keywords:** Laparoscopic cholecystectomy, Exploratory laparotomy, Esophagogastroduodenoscopy, Nonsurgical pneumoperitoneum

## Abstract

Idiopathic pneumoperitoneum (IP) cases are rare and presents with varying symptoms, which makes propositions of standard treatments, clinically impracticable. There are limited IP therapies in the literature, necessitating a need, to continually highlight unique cases for the purpose of clinical education and training. This case describes an IP and management of a 34-year-old male who recently underwent a laparoscopic cholecystectomy. Patient presented to the emergency room with recurrent gastrointestinal (GI) symptoms. Despite two negative exploratory laparotomies without confirmatory evidence of GI perforations, the GI symptoms persisted, making it an unusual case. The surgeons elected to a multispecialty approach, detailing patient-specific symptoms, and corresponding treatments of the case. Based on the successful outcome of this patient, detailed knowledge of medical history, repeated physical assessments and patient-specific and comprehensive approach was shown to reduce unnecessary exploratory laparotomy, improved clinical outcomes and decrease in complications.

## INTRODUCTION

Pneumoperitoneum is known to affect all age groups. The etiology and clinical manifestations vary per individuals, and in about 90% of cases associated with visceral perforation are linked with gastric and duodenal ulcer. The other 10% of cases, broadly described as idiopathic pneumoperitoneum (IP) have no direct connection with a demonstrable risk factor [1, 2].

The current literature indicates no established treatments of incidentally detected IP, because of a rarity of cases and vast clinical presentations. However, adequate management of IP requires detailed knowledge of medical history, repeated physical assessments and continued evaluation of individual patients to avoid unnecessary exploratory laparotomy (EL) [3].

Approximately 90% of pneumoperitoneum cases require emergency surgical interventions because of the perforation of the hollow viscus. However, the remaining 10% can be self-limiting, and managed conservatively if there are none existential abdominal viscus perforation or any associations with the alimentary tract perforations [4].

We describe the management of a patient diagnosed with an IP. The patient presented with recurrent gastrointestinal (GI) symptoms despite two negative EL for GI perforations and a recent laparoscopic cholecystectomy (LC).

Treatment course and outcomes are detailed in this case.

## CASE REPORT

A 34-year-old male presented to the emergency room (ER) with a significant history of Type II diabetes mellitus, obesity, adenomatous colon polyp and osteoarthritis of the lumbar spine.

**Figure 1:**
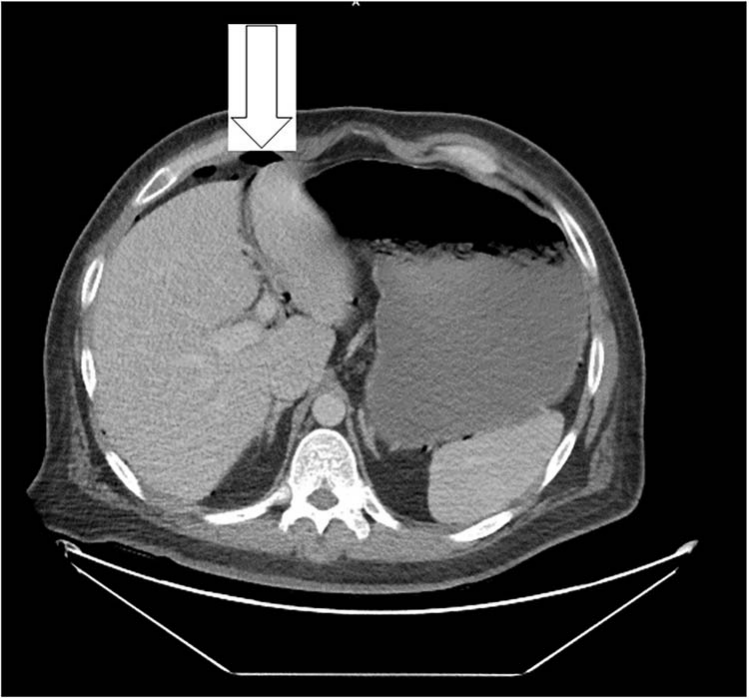
Free peritoneal air is likely related to recent LC.

Ten days prior to ER admission, he presented to an outlying hospital with nausea, vomiting and diarrhea episodes and underwent a LC. A baseline computerized tomography scan (CT) demonstrated pneumoperitoneum (see Fig. 1). Because of the lack of inflammatory changes in the GI tract, viscus perforation was an unlikely consideration. An EL was performed that came back negative and he was subsequently discharged.

**Figure 2:**
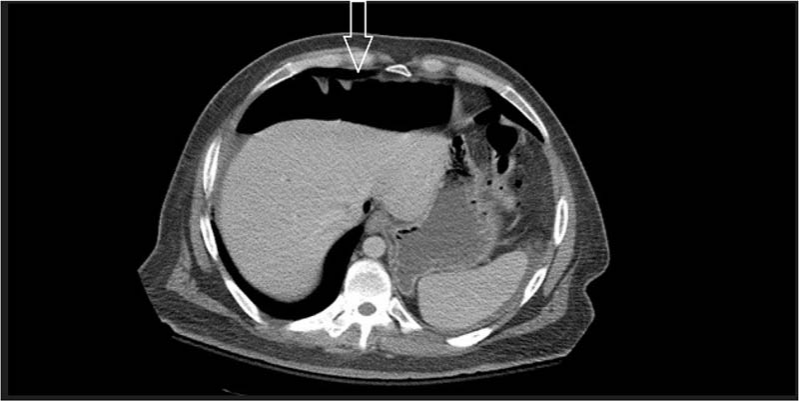
Large amount of free intraperitoneal air is present.

Ten days after discharge, he was readmitted with the same symptoms of nausea and vomiting. The CT showed pneumoperitoneum (see Fig. 2) again with another EL with consequent negative results and no viscus perforations. Lesser amounts of free fluid in both upper quadrants and in the pelvis were noted. These findings remain suspicious for a perforated viscus. Few days later, the patient reported feeling better and was discharged.

**Figure 3:**
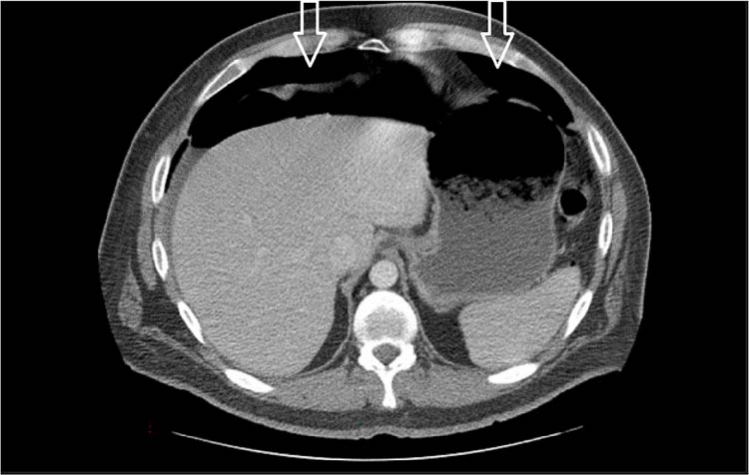
Amount of pneumoperitoneum increased compared with previous CTs.

Eleven days post-discharge, he presented to the ER with the same symptoms. CT once again demonstrated pneumoperitoneum (see Fig. 3). Perforated hollow viscus structure is suspected; however, no rim-enhancing abscess is evident. Given his previous two negative ex-laps, a repeat EL was deferred. A conservative treatment plan of pneumoperitoneum was implemented for IP.

Continued observation and GI consult for an esophagogastroduodenoscopy demonstrated findings consistent with gastroparesis. A small bowel follow through was completed without any contrast extravasation or any abnormalities.

Physical and a complete blood count exams with differentials were within normal range.

The patient stated taking MiraLAX and Dulcolax regularly while consuming an appropriate amount of water daily with minimal improvements.

**Figure 4:**
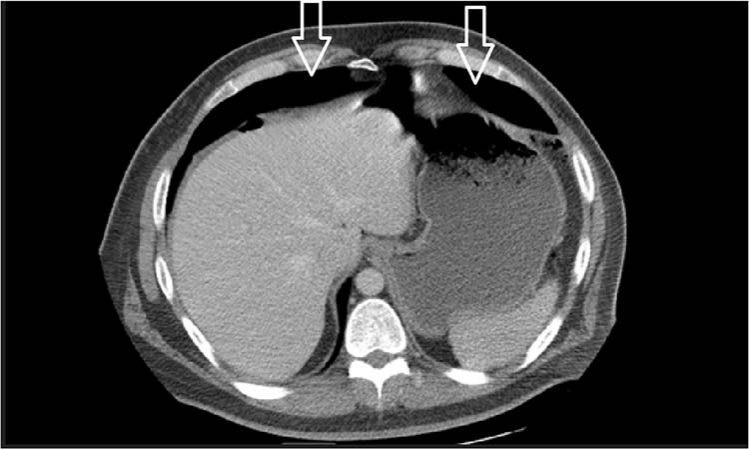
Similar appearance of moderate volume pneumoperitoneum compared with last CT.

Dietary consultation started him on a gastroparesis diet where he progressed well. Upon hospital discharge CT showed a moderate volume of pneumoperitoneum with a small volume of perihepatic and perisplenic ascites. No areas of focal bowel wall thickening were noted (see Fig. 4).

During the follow-up clinic visit after 23 days of discharge, the patient reported he has returned to work fully, and has been compliant with the lifting restrictions. Furthermore, the patient adhered strictly to prescribed gastroparesis diet which included abstaining from beef and pork. He maintained a bowel regimen of Reglan and MiraLAX, which significantly improved symptoms. Skin incision healed well except for a blister noted at the upper portion of the incision.

## DISCUSSION

IP is quite rare and to the best of our knowledge not adequately reported in the literature. In cases where pneumoperitoneum is nonsurgical or idiopathic, there has been unnecessary explorative laparotomies, typically causing more harm than benefits [1, 5]. The current literature suggests if a patient does not have any peritoneal symptoms and if imaging examinations show absence of abdominal cavity pathology, then, clinicians may consider spontaneous pneumoperitoneum, which does not require surgical intervention [4].

Traditionally, IP diagnosis is concluded after surgical and nonsurgical pneumoperitoneum etiologies have been ruled out. This is a rare case of persistent IP that presented with GI symptoms following a LC, without confirmatory evidence of GI perforation on contrast enhanced abdominal CT and two negative outcomes of exploratory laparotomies. Highlighting such unique cases for the purpose of clinical education helps raise awareness of holistic and multispecialty approach in the management of the varying IP presentations.

## CONFLICT OF INTEREST STATEMENT

None declared.

## FUNDING

None.
